# Optimization of parameters in cylindrical and surface grinding for improved surface finish

**DOI:** 10.1098/rsos.171906

**Published:** 2018-05-30

**Authors:** Dinesh Kumar Patel, Deepam Goyal, B. S. Pabla

**Affiliations:** Department of Mechanical Engineering, National Institute of Technical Teachers Training and Research, Sector-26, Chandigarh 160019, India

**Keywords:** cylindrical grinding, optimization, grinding parameters, EN8 steel

## Abstract

Surface integrity has attracted the attention of researchers for improving the functional performance of engineering products. Improvement in surface finish, one of the important parameters in surface integrity, has been attempted by researchers through different processes. Grinding has been widely used for final machining of components requiring smooth surfaces coupled with precise tolerances. Proper selection of grinding wheel material and grade with grinding parameters can result in an improved surface finish and improved surface characteristics. The present work reports the study of the effect of grinding parameters on surface finish of EN8 steel. Experiments were performed on surface grinding and cylindrical grinding for optimization of grinding process parameters for improved surface finish. Grinding wheel speed, depth of cut, table feed, grinding wheel material and table travel speed for surface grinding operation, and work speed for cylindrical grinding operation were taken as the input parameters with four types of grinding wheels (Al_2_O_3_ of grades K and L, and white alumina of grades J and K). The surface roughness was taken as an output parameter for experimentation. The grinding wheel material and grade have been observed to be the most significant variables for both cylindrical grinding and surface grinding. Surface roughness in the case of surface grinding is better compared to that of cylindrical grinding, which can be attributed to vibrations produced in the cylindrical grinding attachment. Surface roughness (*R*_a_) values of 0.757 µm in cylindrical grinding and 0.66 µm in surface grinding have been achieved.

## 1. Introduction

The manufacturing industry focuses on workpiece dimensional accuracy and surface finish for improved functioning of the machined parts. Surface texture, defined in terms of surface roughness, waviness, lay and flaws, is concerned with the geometric irregularities of the surface of a workpiece. Surface integrity coupled with economical production/processes is the basic requirement of industry [1,2]. Surface roughness influences the performance of mechanical parts and their costs as it affects factors such as friction, ease of holding lubricant, electrical and thermal conductivity and geometric tolerances. There is no comprehensive model that can predict roughness over a wide range of operating conditions. As the surface finish is governed by many factors, its experimental determination is laborious and time-consuming [3]. Grinding has been widely used for final machining of components requiring smooth surfaces coupled with precise tolerances. Proper selection of grinding wheel material and grade and grinding parameters can result in generation of a smooth surface, resulting in improving the surface quality. Many authors have analysed grinding input parameters (wheel speed, table speed, depth of cut and the dressing mode) to study the surface roughness and geometric error [4–7]. Rao & Pawar [8] proposed multi-objective optimization of process parameters of the grinding process using various non-traditional optimization techniques such as artificial bee colony, harmony search and simulated annealing algorithms. The objectives considered in their work were production cost, production rate and surface finish subjected to the constraints of thermal damage, wheel wear and machine tool stiffness. The process variables considered for optimization were wheel speed, workpiece speed, depth of dressing and lead of dressing. These variables were also optimized in order to minimize production cost and surface roughness [9]. An evolutionary algorithm has been proposed in which the optimization was introduced in Pareto's sense; all acceptable and non-dominated solutions were remembered; therefore, the final result was not a single solution, but a whole set. Krajnik *et al*. [10] described a systematic methodology for empirical modelling and optimization of the plunge centreless grinding process. The model was fully constructed by determination of its structure and regression coefficients. The focus of the study was the determination of an optimum centreless grinding system set-up and operating conditions for minimization of surface roughness. Kwak *et al*. [11] analysed the grinding power spent during the process and the surface roughness of the ground workpiece in the external cylindrical grinding of hardened SCM440 steel using the response surface method. Capello & Semeraro [12] investigated the influence of the depth of cut and the peripheral velocity of the workpiece in cylindrical grinding. The relationship between process parameters and residual stresses was presented. It was concluded that in ‘easy to grind’ conditions, an increase in workpiece velocity leads to an increase in residual stresses, but in ‘difficult to grind’ conditions an increase in workpiece velocity results in a decrease in residual stresses. The generalized models have been constructed in such a way that they can be adapted to different grinding situations by modifying the values of constants and exponents. Thus, the system uses the modern techniques of knowledge engineering and process modelling and brings traditional grinding parameter selection into a complete new and advanced environment [13–16]. Li *et al*. [17] created the precondition for grinding automation, virtual grinding and an intelligent grinding system by computer simulation and an actual grinding process. A scatter search-based optimization approach has been developed to optimize the grinding parameters (i.e. wheel speed, workpiece speed, depth of dressing and lead of dressing) using a multi-objective function model with a weighted approach for the surface grinding process, and results were compared with the results obtained by the ants-colony algorithm, genetic algorithm and quadratic programming techniques [18]. Analysis of the literature reveals that the researchers carried out the work either on a surface grinding process or a cylindrical grinding process but comparison has not been reported. Limited work has been reported on evaluation of the surface integrity of EN8 steel after grinding. There is a scope to study the effect of different grinding wheel materials and their grades together on surface finish in grinding operations.

In this paper, the effect of grinding parameters on surface finish for EN8 steel is presented. Experiments were performed on surface grinding and a cylindrical grinding for optimization of grinding process parameters for improved surface finish. The surface roughness was taken as an output parameter for experimentation.

## 2. Methodology

The aim of the experimental work was to compare surface grinding and cylindrical grinding operations to optimize the grinding process parameters for improving the surface finish. Experiments were conducted on a BLOHM SIMPLEX surface grinding machine with a cylindrical grinding attachment.

### 2.1. Selection of workpiece material

The workpiece material used was EN8 steel, which is widely used in industrial applications like engine shafts, spindles, connecting rods, studs and screws due to its good mechanical properties. It is medium carbon steel usually supplied untreated and having good tensile strength. The tensile strength varies in the range of 500–800 N mm^−2^.

### 2.2. Experimental set-up

The experiments were conducted on a surface grinding machine with a cylindrical grinding attachment at different combinations of grinding process parameters. For surface grinding, the machine allowed r.p.m. as well as table feed variations, whereas for cylindrical grinding, only the fixed r.p.m. mechanism was available. The existing fixed r.p.m. mechanism was modified for obtaining r.p.m. variations at three levels.

### 2.3. Selection of grinding process parameters

The grinding wheel speed, grinding wheel grade, depth of cut, grinding wheel material and feed rate are the important parameters that affect the surface finish, which in turn affects the productivity and cost of the component. The grinding parameters used for both surface and cylindrical operations are tabulated in table 1. Four grinding wheels of different materials having different grades (Al_2_O_3_ of grades K and L, and white alumina of grades J and K) were used for conducting the experiments. The grinding process parameters and their values at different levels are taken as per table 2.

**Table 1.** Parameters used for surface and cylindrical operations.

parameters	surface grinding	cylindrical grinding
grinding wheel speed	√	√
depth of cut	√	√
table feed	√	√
grinding wheel material	√	√
workpiece speed		√
table travel speed	√

**Table 2.** Grinding process parameters and their values at different levels.

		levels and values			
process parameters	symbol	1	2	3	4
grinding wheel speed (r.p.m.)	A	1400	2800	—	—
workpiece speed (r.p.m.)	B	128	278	323	656
depth of cut (µm)	C	10	20	30	40
material and grade of grinding wheel	D	A60K5V10	A60L5V10	AA60J5V8	AA46/54K5V8
table cross feed (m min^−1^)	E	0.012	0.018	0.024	0.030
table travel speed (m min^−1^)	F	6	10	14	18

### 2.4. Design of experiments

Experiments have been conducted on the basis of the Taguchi method to categorize the experimentation into four levels for surface grinding and cylindrical grinding process parameters. The L32 Orthogonal Array (OA) was used for the experimental work.

### 2.5. Experimentation

The experiments were conducted on the surface grinding machine with a cylindrical grinding attachment at different combinations of grinding process parameters. Experiments were performed at two values of grinding wheel speed, i.e. 1400 and 2800 r.p.m., for both surface and cylindrical grinding operations. A total of 32 experiments were performed. For each test run, three trials were performed to increase the accuracy of results for a better surface finish result. The average of these three trial values has been used for experimental analysis. A total of 96 test runs were made during experimentation.

### 2.6. Cylindrical grinding

For cylindrical grinding, a round bar 110 mm long and 20.4 mm in diameter was used. Experiments were conducted on the surface grinding machine with a cylindrical grinding attachment by using water-soluble coolant. Grinding wheel speed, depth of cut, table feed, grinding wheel material and work speed for cylindrical grinding operation were taken as the input parameters with four types of grinding wheels (Al_2_O_3_ of grades K and L, and white alumina of grades J and K).

### 2.7. Surface grinding

A flat plate of 132 × 28.5 × 6.15 (L × B × W) mm was used for the surface grinding experiments using the same lubricant. The input parameters used for cylindrical grinding were kept the same, except that the work speed was replaced by the table travel speed for surface grinding operation.

The surface roughness values for all the experiments for surface and cylindrical grinding operations were measured. The signal-to-noise (S/N) ratio and analysis of variance (ANOVA) were used to study the performance characteristics of the grinding operation. Confirmation tests were carried out to compare the results of predicted values with the experimental value. ANOVA was carried out to identify the significant factors affecting the surface roughness.

## 3. Results

The results obtained from experimental work for the optimization of grinding process parameters are given in annexure A. The results obtained from the experimental data are discussed below.

### 3.1. Evaluation of S/N ratios

At each set of input variables, three experiments were conducted and the average of these three trial values has been taken for analysis. The mean surface roughness values and the corresponding S/N ratio of each test run obtained for both grinding operations are shown in annexure A.

#### 3.1.1. Level mean response analysis

The level mean values of S/N ratios calculated for four levels of grinding parameters of surface grinding and cylindrical grinding operations are as shown in annexure A. The level mean response S/N ratios help in analysing the trend of the quality characteristics with respect to the variation of the grinding input parameters. The level mean response plots based on the S/N ratios are used in optimizing the surface roughness.

The rank of grinding process parameters used in both grinding processes is given in table 3 based on S/N ratios. Delta, the value calculated for ranking the grinding process parameters, was used in both the grinding processes. The value of *λ* was calculated by taking the difference in the maximum value from the minimum value of S/N ratios. The parameter having a larger difference of S/N ratios is ranked first; similarly, other differences were compared and ranked accordingly.

**Table 3.** Level mean of S/N ratios for cylindrical and surface grinding operations.

level mean for cylindrical grinding					
level	A	B	C	D	E
1	−2.09713	−2.23815	−2.35052	0.12759	−2.40066
2	−2.30429	−0.94795	−2.98385	0.38327	−2.19290
3	—	−2.36117	−1.58109	−4.89409	−2.16073
4	—	−3.25558	−1.88738	−4.31962	−2.04855
*λ*	0.20716	2.30763	1.40277	5.27736	0.35210
rank	5	2	3	1	4

level mean for surface grinding					
level	A	F	C	D	E
1	−3.12268	−3.06506	−2.95014	0.99695	−3.53034
2	−3.57579	−2.12479	−3.79640	0.70567	−4.11499
3	—	−3.93171	−3.39267	−7.84494	−3.21745
4	—	−4.27539	−3.25774	−7.25463	−2.53416
*λ*	0.45311	2.15060	0.84626	8.84189	1.58083
rank	5	2	4	1	3

##### 3.1.1.1. Cylindrical grinding

**Figure 1.**
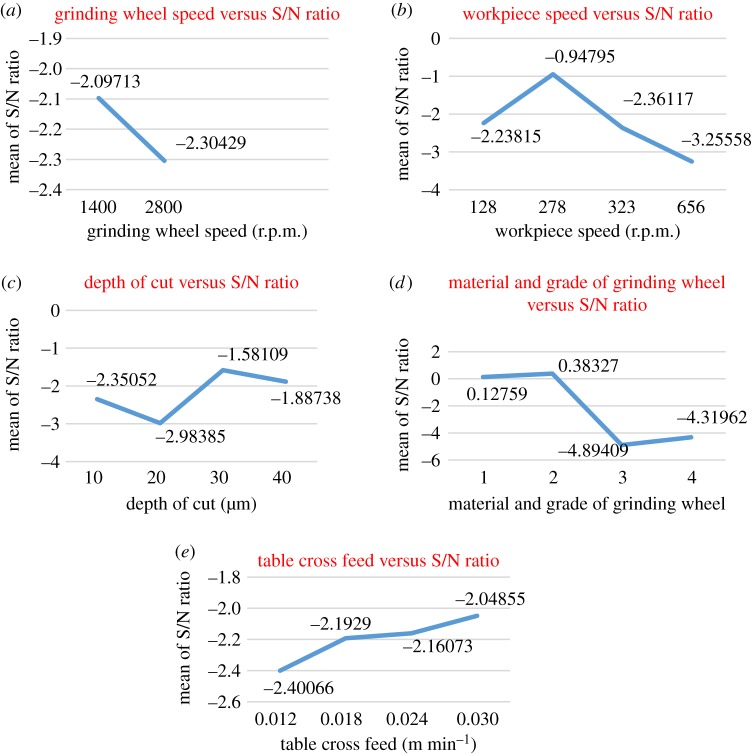
Level average response graphs for quality characteristic in cylindrical grinding based on the S/N ratio.

The level mean response plots for various quality characteristics based on the S/N ratios in cylindrical grinding are shown in figure 1*a–e*. Figure 1*a* shows that the S/N ratio corresponding to 1400 r.p.m. of grinding wheel was larger, which is desirable for a better surface finish. The S/N ratio corresponding to 2800 r.p.m. of the grinding wheel was lower. Figure 1*b* shows the variation of S/N ratios for workpiece speed. It has been noted that the S/N ratio corresponding to 278 r.p.m. of workpiece speed was larger, which is advantageous for achieving a good surface finish. The S/N ratio at the 656 r.p.m. workpiece speed was the lowest. Also, the S/N ratio increases slightly as the workpiece speed is increased from 128 to 278 r.p.m.; then, the S/N ratio decreases constantly on further increasing the workpiece speed. Figure 1*c* shows the graph of S/N ratios for different depths of cut. It has been observed that the S/N ratio corresponding to the 30 µm depth of cut was larger, which signifies improved surface finish. The S/N ratio at the 20 µm depth of cut was the lowest. Initially, the S/N ratio decreases from the 10 µm to the 20 µm depth of cut, then it increases slowly for higher values of depth of cut. The graphs in figure 1*b* and figure 1*d* depict similar trend in the effect of workpiece speed and material and grade of grinding wheel on S/N ratio, respectively. The S/N ratio corresponding to the second grinding wheel was found to be larger, which is desirable for a better surface finish. The S/N ratio corresponding to the third grinding wheel was the lowest. It has been concluded that the aluminium oxide grinding wheel gave better performance than the white alumina grinding wheel. It is found from figure 1*e* that the S/N ratio increases as the table cross feed increases. The surface finish is better at higher values of table cross feed. The graph shows almost the same trend as that of the depth of cut.

##### 3.1.1.2. Surface grinding

**Figure 2.**
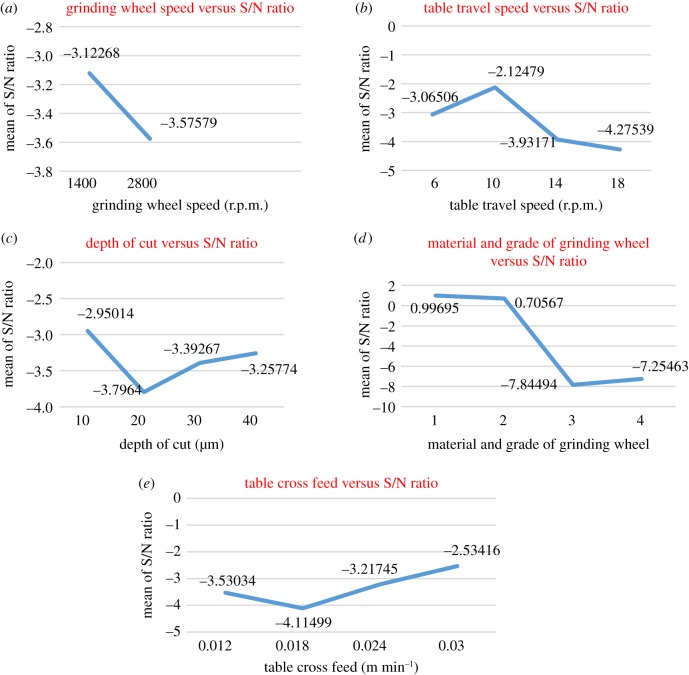
Level average response graphs for quality characteristic in surface grinding based on the S/N ratio.

The level mean response plots for various quality characteristics in surface grinding based on the S/N ratios are shown in figure 2*a*–*e*. In figure 2*a*, the S/N ratio corresponding to 1400 r.p.m. of the grinding wheel is larger, which is desirable for a better surface finish. The S/N ratio corresponding to 2800 r.p.m. of grinding wheel was lower. Figure 2*b* shows the graph of S/N ratios for table travel speed. The value of the S/N ratio at 10 m min^−1^ of table travel speed was the largest, which is desirable for a better surface finish. The value of the S/N ratio at 18 µm of depth of cut was the lowest. The S/N ratio decreases as the table travel speed is either increased or decreased from 100 m min^−1^. Figure 2*c* shows the graph of S/N ratios with respect to depth of cut. The S/N ratio corresponding to 10 µm of depth of cut was larger, which is desirable for a better surface finish. The S/N ratio at 20 µm of depth of cut was the lowest. The S/N ratio initially decreases from the 10 µm to the 20 µm depth of cut, after which it increases slowly for higher values of the depth of cut. It is concluded from figure 2*d* that the aluminium oxide grinding wheel gave better performance than the white alumina grinding wheel. It was also observed from the graph that the S/N ratio corresponding to the first grinding wheel was larger, which is desirable for a better surface finish. The S/N ratio corresponding to the third grinding wheel was the lowest. It is observed from figure 2*e* that the value of the S/N ratio was highest at the 0.03 m min^−1^ table cross feed. Initially, the S/N ratios decrease with increase in table cross feed, but after the 0.018 m min^−1^ cross feed, the S/N ratio increases slowly. The surface finish is better at higher values of table cross feed. The graph shows the same trend as that of the depth of cut.

### 3.2. Analysis of variance

Table 4 shows the relative effect of each controlled parameter on surface roughness in both the categories of grinding using S/N ratio-based ANOVA for a significance level of *a* = 0.05 (confidence level of 95%). The material and grade of grinding wheel is found to be the prominent factor influencing the surface roughness in cylindrical grinding with 70.76% of contribution. The other variables that have an effect on surface roughness (*R*_a_) are grinding wheel speed (8.2%), depth of cut (3.38%), table cross feed (0.19%) and grinding wheel speed (0.13%) for cylindrical grinding. The error contribution was 17.33%.

**Table 4.** ANOVA for S/N ratios in different grinding processes (95% confidence level). Note: d.f., degrees of freedom; SS, sum of squared deviations; *F*-value, *F*-test value (at least 95% confidence); *p*-value, probability level (less than 0.05); PC, percentage contribution.

cylindrical grinding						
source	d.f.	adjacent SS	adjacent MS	*F*-value	*p*-value	PC (%)
A	1	0.343	0.343	0.13	0.718	0.13
B	3	21.674	7.225	2.84	0.067	8.20
C	3	8.943	2.981	1.17	0.348	3.38
D	3	187.091	62.364	24.50	0.000	70.76
E	3	0.518	0.173	0.07	0.976	0.19
error	18	45.813	2.545	—	—	17.33
total	31	264.382	—	—	—	—

surface grinding						
source	d.f.	adjacent SS	adjacent MS	*F*-value	*p*-value	PC (%)
A	1	1.642	1.642	0.53	0.476	0.25
F	3	22.216	7.405	2.39	0.103	3.37
C	3	2.956	0.985	0.32	0.813	0.45
D	3	566.360	188.787	60.81	0.000	85.88
E	3	10.407	3.469	1.12	0.368	1.58
error	18	55.881	3.104	—	—	8.47
total	31	659.463	—	—	—	—

For surface grinding, the results of ANOVA depict that the *F*-value for the parameter, grinding wheel material and grade dominates all other input parameters with an 85.88% contribution. The other variables that have an effect on surface roughness are table travel speed (3.37%), table cross feed (1.58%), table cross feed (1.58%), depth of cut (0.45%) and grinding wheel speed (0.25%). The error contribution was 8.47%. Table 4 shows that the depth of cut and the grinding wheel speed are the least significant parameters in surface grinding and their contribution is negligible.

## 4. Mathematical modelling

After obtaining the optimal level of design parameters, the final step is to predict and verify the surface roughness using the optimal level of the design parameters.

### 4.1. Cylindrical grinding

At optimum setting conditions, S/N ratios of cylindrical grinding were determined by using the following equation:

4.1 $$\eta_{G}\;=\;{\bar{\eta }}_{G}\;+\;(\bar{A_{0}}-\bar{\eta_{G}})+\;(\bar{B_{0}}-\bar{\eta_{G}})\;+\;(\bar{C_{0}}-\bar{\eta_{G}})\;+\;(\bar{D_{0}}-\bar{\eta_{G}})\;+\;(\bar{E_{0}}-\bar{\eta_{G}}),$$

where $\eta_{G}$ is the S/N ratio calculated at the optimum levels and ${\bar{\eta }}_{G}$ is the mean S/N ratios of all parameters; and ${\bar{A}}_{0}$ (−2.09713), ${\bar{B}}_{0}$ (−0.94795), ${\bar{C}}_{0}$ (−1.58109), ${\bar{D}}_{0}$ (0.38327) and ${\bar{E}}_{0}$ (−2.04855) are the mean S/N ratio values for A, B, C, D and E parameters, respectively, at the mean S/N ratio when these parameters are at the optimum surface roughness level. The predicted S/N ratio of 2.51139 dB is transformed to *R*_a_ = 0.748 µm.

### 4.2. Surface grinding

At optimum setting conditions, the S/N ratios of surface grinding were determined by using the following equation:

4.2 $$\eta_{G}\;=\;{\bar{\eta }}_{G}\;+\;(\bar{A_{0}}-\bar{\eta_{G}})+\;(\bar{F_{0}}-\bar{\eta_{G}})\;+\;(\bar{C_{0}}-\bar{\eta_{G}})\;+\;(\bar{D_{0}}-\bar{\eta_{G}})\;+\;(\bar{E_{0}}-\bar{\eta_{G}}),$$

where ${\bar{A}}_{0}$ (−2.09713), ${\bar{F}}_{0}$ (−0.94795), ${\bar{C}}_{0}$ (−1.58109), ${\bar{D}}_{0}$ (0.38327) and ${\bar{E}}_{0}$ (−2.04855) are the S/N ratio values for A, F, C, D and E parameters, respectively, at the mean S/N ratio when these parameters are at the optimum surface roughness level. The predicted S/N ratio of 3.662124 dB is transformed to *R*_a_ = 0.655 µm.

## 5. Confirmation test

The predicted optimum surface roughness value of means was validated using a confirmation test. The confirmation experiments were performed at the optimum variable level (i.e. grinding wheel speed = 1400 r.p.m., workpiece speed = 278 r.p.m., depth of cut = 30 µm, grinding wheel material and grade = A60L5V10, table cross feed = 0.03 m min^−1^) for cylindrical grinding. Three tests were conducted at the same optimum parameter values for both the grinding operations. The values of surface roughness measured are given in table 5. The mean of these three surface roughness values was taken to compare the predicted surface roughness. Similarly, the confirmation test for surface grinding was performed at the optimum level of parameters (i.e. grinding wheel speed = 1400 r.p.m., table travel speed = 10 m min^−1^, depth of cut = 10 µm, grinding wheel material and grade = A60K5V10 and table cross feed = 0.03 m min^−1^). The percentage error between predicted roughness and experimental roughness is 1.20% and 0.75% for cylindrical grinding and surface grinding, respectively. The overall results obtained from the experiments have been analysed and compared in table 6.

**Table 5.** Results of the confirmation test for surface roughness.

cylindrical grinding					roughness					
A	B	C	D	E	test 1	test 2	test 3	mean	predicted value	error (%)
1400	278	30	A60L5V10	0.03	0.78	0.74	0.75	0.757	0.748	1.2

surface grinding					roughness					
A	F	C	D	E	test 1	test 2	test 3	mean	predicted value	error (%)
1400	10	10	A60K5V10	0.03	0.68	0.65	0.65	0.66	0.655	0.75

**Table 6.** Comparison of results obtained during cylindrical and surface grinding operations.

no.	cylindrical grinding	surface grinding
1	The predicted surface roughness for cylindrical grinding was evaluated as 0.748 µm.	The predicted surface roughness for surface grinding was evaluated as 0.660 µm.
2	The percentage error is approximately 1.2%.	The percentage error is 0.75%.
3	The grinding wheel material and grade greatly influence the surface roughness, followed by the workpiece speed (r.p.m.), depth of cut (µm), table cross feed (m min^−1^) and grinding wheel speed (r.p.m.) for cylindrical grinding operation.	The grinding wheel material and grade greatly influence the surface roughness, followed by table travel speed (m min^−1^), table cross feed (m min^−1^), depth of cut (µm) and grinding wheel speed (r.p.m.) in surface grinding operation.
4	The optimum conditions of parameters for lower surface roughness for EN8 steel were the grinding wheel speed 1400 r.p.m., workpiece speed 278 r.p.m., depth of cut 30 µm, grinding wheel A60L5V10 and table cross feed 0.03 m min^−1^.	The optimum conditions of grinding parameters for lower surface roughness for EN8 steel were the grinding wheel speed 1400 r.p.m., table travel speed 10 m min^−1^, depth of cut 10 µm, grinding wheel A60K5V10 and table cross feed 0.03 m min^−1^ for surface grinding.
5	The percentage contributions of the grinding parameters: material and grade of grinding wheel (70.76%), grinding wheel speed (8.2%), depth of cut (3.38%), table cross feed (0.19%) and grinding wheel speed (0.13%).	The percentage contributions of the grinding parameters: material and grade of grinding wheel (85.88%), table travel speed (3.37%), table cross feed (1.58%), depth of cut (0.45%) and grinding wheel speed (0.25%).

## 6. Conclusion

In this paper, the effect of grinding parameters on surface finish on EN8 steel has been analysed. Experiments were performed on surface and cylindrical grinding for optimization of grinding process parameters for improved surface finish. The following conclusions have been obtained from the experimentation and analysis of results:

(a) Surface roughness in the case of surface grinding is better when compared with cylindrical grinding.
(b) The predicted surface roughness (*R*_a_) for cylindrical and surface grinding was evaluated to be 0.748 µm and 0.660 µm, whereas the roughness value from the confirmation experiments for both operations was 0.757 µm and 0.655 µm, respectively. The percentage error of surface roughness for cylindrical and surface grinding is found to be 1.2% and 0.75%, respectively.
(c) The material and grade of the grinding wheel have been found to be most prominent factors influencing surface roughness for both grinding operations.
(d) The optimum conditions of cylindrical and surface grinding parameters for lower surface roughness for EN8 steel have been determined.

As the conclusions are based on the effect of a set of input parameters on surface roughness, it is expected that the proposed parameters will give the desired results on any other grinding machine with controlled vibrations, which can affect the results significantly.

## Supplementary Material

Supplementary material

## Data accessibility

Our data are deposited at Dryad: http://dx.doi.org/10.5061/dryad.pr7kk52 [19].

## Appendix A

Annexure A (Experimental results for response and their S/N ratios).

**Table:**

cylindrical grinding						surface grinding					
	test results of surface roughness (µm)						test results of surface roughness (µm)				
test run no.	trial 1	trial 2	trial 3	mean surface roughness (µm), *R*_a_	S/N ratios (dB)	test run no.	trial 1	trial 2	trial 3	mean surface roughness (µm), *R*_a_	S/N ratios (dB)
1	0.89	0.86	0.92	0.89000	1.01220	1	0.69	0.64	0.63	0.65333	3.69735
2	0.95	1.03	0.89	0.95667	0.38476	2	0.86	0.87	1.03	0.92000	0.72424
3	1.76	1.57	1.71	1.68000	−4.50619	3	2.59	2.95	2.71	2.75000	−8.78665
4	1.69	1.89	1.95	1.84333	−5.31206	4	2.10	2.33	2.05	2.16000	−6.68908
5	0.87	0.97	0.85	0.89667	0.94735	5	0.86	0.87	1.10	0.94333	0.50673
6	1.11	1.09	1.21	1.13667	−1.11269	6	1.06	1.00	1.02	1.02667	−0.22862
7	1.05	1.12	0.98	1.05000	−0.42379	7	1.21	1.40	1.43	1.34667	−2.58522
8	1.42	1.20	1.44	1.35333	−2.62807	8	1.65	1.82	1.74	1.73667	−4.79435
9	1.18	1.19	1.14	1.17000	−1.36372	9	0.97	1.02	0.95	0.98000	0.17548
10	0.88	1.02	0.95	0.95000	0.44553	10	1.04	1.07	0.98	1.03000	−0.25674
11	1.95	1.78	1.68	1.80333	−5.12150	11	2.68	2.43	2.52	2.54333	−8.10805
12	1.82	1.56	1.66	1.68000	−4.50619	12	2.85	2.75	2.92	2.84000	−9.06637
13	1.03	1.08	1.11	1.07333	−0.61467	13	0.084	0.91	0.89	0.62800	4.04081
14	1.11	1.09	1.15	1.11667	−0.95850	14	1.06	1.00	1.02	1.02667	−0.22862
15	1.74	1.62	1.76	1.70667	−4.64299	15	2.87	2.85	2.79	2.83667	−9.05618
16	1.80	1.78	1.85	1.81000	−5.15357	16	2.96	2.89	2.91	2.92000	−9.30766
17	1.63	1.62	1.83	1.69333	−4.57483	17	1.92	2.17	2.25	2.11333	−6.49935
18	1.93	1.96	2.08	1.99000	−5.97706	18	2.38	2.73	2.42	2.51000	−7.99347
19	0.75	0.85	0.79	0.79667	1.97443	19	0.96	0.89	0.87	0.90667	0.85102
20	1.11	1.02	1.20	1.11000	−0.90646	20	1.01	0.98	0.95	0.98000	0.17548
21	1.21	1.28	1.19	1.22667	−1.77455	21	2.24	2.66	2.40	2.43333	−7.72402
22	1.73	2.09	2.27	2.03000	−6.14992	22	2.36	2.33	2.20	2.29667	−7.22197
23	0.77	0.78	0.84	0.79667	1.97443	23	0.78	0.90	1.07	0.91667	0.75574
24	0.79	0.82	0.89	0.83333	1.58366	24	0.59	0.66	0.58	0.61000	4.29340
25	2.06	2.17	2.07	2.10000	−6.44439	25	2.99	2.89	2.84	2.90667	−9.26791
26	1.94	1.89	1.86	1.89667	−5.55984	26	2.27	2.31	2.28	2.28667	−7.18407
27	0.71	0.85	0.86	0.80667	1.86608	27	0.86	0.87	0.89	0.87333	1.17643
28	0.82	0.77	0.85	0.81333	1.79466	28	0.87	0.88	0.90	0.88333	1.07754
29	2.23	2.08	2.13	1.99333	−5.99158	29	2.41	2.89	2.71	2.67000	−8.53023
30	1.89	1.64	1.77	1.76667	−4.94311	30	2.39	2.52	2.61	2.50667	−7.98194
31	1.56	1.42	1.65	1.54333	−3.76918	31	1.09	1.24	1.19	1.17333	−1.38840
32	0.92	1.02	1.05	0.99667	0.02897	32	1.12	1.34	1.21	1.22333	−1.75087
